# Proof-of-concept MALDI-TOF-MS assay for the detection of Toxin B enzymatic activity in *Clostridioides difficile* infection

**DOI:** 10.1128/spectrum.02453-24

**Published:** 2025-03-31

**Authors:** Josef Dvorak, Lukáš Fojtík, Ljubina Adámková, Katerina Vlkova, Vendula Studentova, Katerina Chudejova, Lenka Geigerová, Michael Volny, Petr Novak, Jaroslav Hrabak, Petr Pompach

**Affiliations:** 1 Institute of Microbiology of the Czech Academy of Sciences, BIOCEV, Vestec, Czechia; 2 Department of Biochemistry, Charles University, Prague, Czechia; 3 Biomedical Center, Faculty of Medicine in Pilsen, Charles University, Pilsen, Czechia; 4 Department of Microbiology, Faculty of Medicine in Pilsen, Charles University, Pilsen, Czechia; 5 Department of Analytical Chemistry, University of Chemistry and Technology, Prague, Czechia; 6 Institute of Biotechnology of the Czech Academy of Sciences, BIOCEV, Vestec, Czechia; Tainan Hospital, Ministry of Health and Welfare, Tainan, Taiwan

**Keywords:** *C. difficile*, Toxin B, MALDI-TOF-MS, diagnostics

## Abstract

Matrix-assisted laser desorption/ionization time-of-flight (MALDI-TOF) mass spectrometers have become an integral part of all modern clinical microbiology laboratories. They serve as the key tool for pathogen identification and antibiotic resistance determination. However, certain limiting conditions must be fulfilled. The pathogen cannot be tested directly from the sample and requires the cultivation of a pure colony, which means that the standard protocol takes additional time, workforce, and consumables. The testing protocol is also more complicated when it comes to anaerobes. In our work, we focused on the functional detection of *Clostridioides difficile*, an important nosocomial human pathogen that is responsible for diarrhea and can lead to life-threatening colitis, as a model diagnostic problem. The virulence of *C. difficile* is mainly caused by two toxins, Toxin A and Toxin B. Established diagnostic methods, including nucleic acid amplification testing methods and immunoassays, detect the presence of the microorganism or the presence and concentration of the toxins, with limited ability to gauge infection severity based on the actual biochemical activity of the toxins and thus their potency to cause harm. This work presents proof-of-concept assays that indirectly determine the toxin activity in the human stool, a very complex matrix sample, using the natural RhoA protein as substrate. The RhoA protein substrate was recombinantly prepared with biotin tag modification, which allows its attachment to the NeutrAvidin MALDI chips. In the assay, the RhoA substrate anchored on the MALDI chip undergoes enzymatic glycosylation when exposed to the Toxin B in the stool sample, and the reaction product is then detected by MALDI-TOF mass spectrometry directly from the MALDI chip. The entire assay, from sampling to final mass spectrometry detection, was performed *in situ*, on the NeutrAvidin MALDI chip, which was prepared by unique surface modification technology also described in this work. The assay was successfully tested for the detection of Toxin B in a cohort of patient samples as well as in cell culture of *C. difficile*.

**IMPORTANCE:**

The diagnostics of *Clostridioides difficile* infection is usually based on the identification of the bacterial pathogen and/or on the detection of the Toxins A and B. Due to the variance in Toxins A and B activity across species, the toxin concentration determined by standard methods does not necessarily correlate with the severity of the disease. Assays that would target toxins’ enzymatic activity are not routinely used because the requirements are unsuitable for clinical laboratories. In this study, we demonstrate a new approach that determines the presence and potency of Toxin B indirectly by determining its enzymatic activity rather than its concentration. This is performed by detecting mass difference due to glycosylation of RhoA substrate by Toxin B, which is then determined by matrix-assisted laser desorption/ionization time-of-flight mass spectrometry. The presented proof-of-concept assay thus offers the possibility to quickly determine the activity of *C. difficile* toxins directly in the stool samples without pathogen cultivation.

## INTRODUCTION

During the last decade, matrix-assisted laser desorption/ionization time-of-flight mass spectrometry (MALDI-TOF-MS) has become a standard instrument in clinical microbiology laboratories. MALDI biotyping is a common tool for quick pathogen identification and strain classification based on measuring fingerprint mass spectra of lysed bacterial cells, including strain classification of *Clostridioides difficile* (1, 2). It is a cost-effective, reproducible, and reliable method for the rapid identification of bacteria and fungi (3). Due to its accuracy and the small number of bacteria required, the MALDI-TOF-MS has become the primary tool for identifying bacterial infections. However, in most cases, biotyping still requires cultivation, which is a drawback for some bacteria (4). In some cases, anaerobic cultivation needs additional steps and resources, such as specialized consumables, equipment, or enrichment prior to cultivation (5). MALDI-TOF-MS has also become one of the key inventions that allow the concept of syndrome-based pathogen detection that shapes the current clinical microbiology and personalized medicine. This approach is based on multiplex testing of specific panels in the context of the patient’s symptoms (6). However, gastrointestinal tract pathogens, which are mostly anaerobic, require a more complicated cultivation protocol that limits the use of MALDI-TOF-MS. Thus, personalized medicine assays that target anaerobic species are mostly based on molecular-genetic techniques (e.g., multiplex PCR) (7).

We focused our proof-of-concept study on a clinically important severe nosocomial gut pathogen, *C. difficile*, as a model for the method optimization and demonstration of its potential clinical utility. *C. difficile* is a Gram-positive, strictly anaerobic, spore-forming gut bacterium that requires time-consuming and laborious handling (8, 9). Therefore, their anaerobic cultivation does not align with the modern clinical laboratory demands for low-cost tests and time efficiency (5, 8, 10). The *C. difficile* infection (CDI) provides a heterogeneous clinical picture from asymptomatic carriage and mild or moderate diarrhea to colitis that can result in colon perforation, leading to sepsis and possible death (10–12). The spores persist even in extreme antibacterial healthcare conditions, e.g., heat or common disinfectants, which make CDI a leading cause of healthcare-associated infection, with almost half a million cases per year, leading to nearly 30,000 deaths. In Europe, the number of CDI cases is around 120,000 per year, with 3% attributable mortality (10, 13). A fast method for detecting the CDI in patients while evaluating the potential severity of the infection is thus crucial to provide optimal care, which would increase the patient’s survival rate (11, 13, 14).

The nucleic acid amplification testing (NAAT) methods provide rapid, quantitative data on anaerobic species and are commonly used in determining a CDI presence as a part of the recommended workflows (10, 15). The first step in one of the recommended workflows of CDI identification is the immunoassay (IA) test for glutamate dehydrogenase (GDH), which identifies a possible non-specific infection in the colon. This is followed by NAAT or IA aiming directly at the presence of *C. difficile*. In some cases, the GDH test is simultaneously performed with the IA test for Toxin A and Toxin B. If these tests have inconclusive results, additional testing is performed using a different type of test than previously used. However, the actual algorithms for diagnosing *C. difficile* vary between laboratories (16). More laborious tests to confirm CDI in case of inconclusive results are toxigenic culture (TC) or stool cytotoxicity assay (CTA), which are usually considered to be optional only and are not frequently requested. Another possibility to decide cases with inconclusive results from the laboratory testing is colonoscopy (10, 17, 18).

The NAAT and IA tests are used to detect the presence and concentration of the main virulence factors responsible for the *C. difficile* potency—Toxin A and Toxin B (10, 12, 18). Both these toxins enzymatically modify small Rho GTPases by glycosylation. RhoA protein is the most common target of this modification—its Thr37 is modified by adding glucose from the UDP-glucose substrate. This modification results in RhoA inactivation. Since RhoA participates in several cell processes, including cytoskeleton regulation and apoptosis, its inactivation during colitis may lead to cytoskeleton disorganization and cell death, which, on the macroscale, can result in the perforation of the colon (19, 20).

Importantly, *C. difficile* strains differ in toxin activity. For instance, the strain RT017, which was responsible for outbreaks of CDI in several countries around the world, is missing a gene for Toxin A and only exhibits Toxin B activity (21). Because of the difference among strains, the detection by standard laboratory assays (IA and NAAT) lacks the ability to estimate infection severity (18, 21, 22), which calls for assays based on the activity of the toxins. Another diagnostic complication arises from the fact that the toxins can be produced by other bacteria, e.g., *Paeniclostridium sordellii,* due to the virulence transfer. This is a result of the fact that the virulence genes, including those encoding for toxins, can also be spread in microbial populations by horizontal gene transfer (23–25). That is why assaying the actual toxin activity can improve the accuracy of the diagnosis by adding complementary information orthogonal to standard methods. As mentioned, there are two diagnostic methods, TC and CTA, that can evaluate pathogen potency based on the cytotoxic activity (7, 11, 14). However, they are not routinely used because they are time-consuming, laborious, and require experienced workers and specialized equipment (10).

This work demonstrates a proof-of-concept assay that can screen Toxin B enzymatic activity using the same MALDI-TOF mass spectrometers that are already available in clinical microbiological laboratories. The assay can be done in complex human samples without a need for cultivation. The detection assay presented here was based on monitoring the enzymatic activity of Toxin B on the affinity-tagged (by biotinylation) recombinant RhoA, which was prepared in-house using BirA-producing *Escherichia coli* (26). The substrate underwent an enzymatic modification *in situ* on the surface of the NeutrAvidin MALDI chips by Toxin B in the stool sample. The NeutrAvidin chips were manufactured using ambient ion soft landing technology. This is a unique surface modification technique that allows the attachment of protein molecules to different inert surfaces with the retention of the protein’s original biological activity (27–30). The immobilized RhoA substrate was modified by glucose addition, and the mass difference was then detected by MALDI-TOF-MS. The described MALDI assay was optimized and tested using the stool samples of a cohort of CDI-suspected patients. The importance of this proof-of-concept assay needs to be seen in the context of a recent study that shows that the potency of CDI does not directly correlate with the concentration of the toxin in the stool (31). That is why a fast instrumentation-based assay that can measure the actual activity of the toxins instead of concentration provides important clinical information complementary to standard techniques while avoiding the laborious cultivation and CTA or TC approaches.

## RESULTS

### Fast protein liquid chromatography (FPLC) purification and mass spectra of RhoA substrate

**Fig 1:**
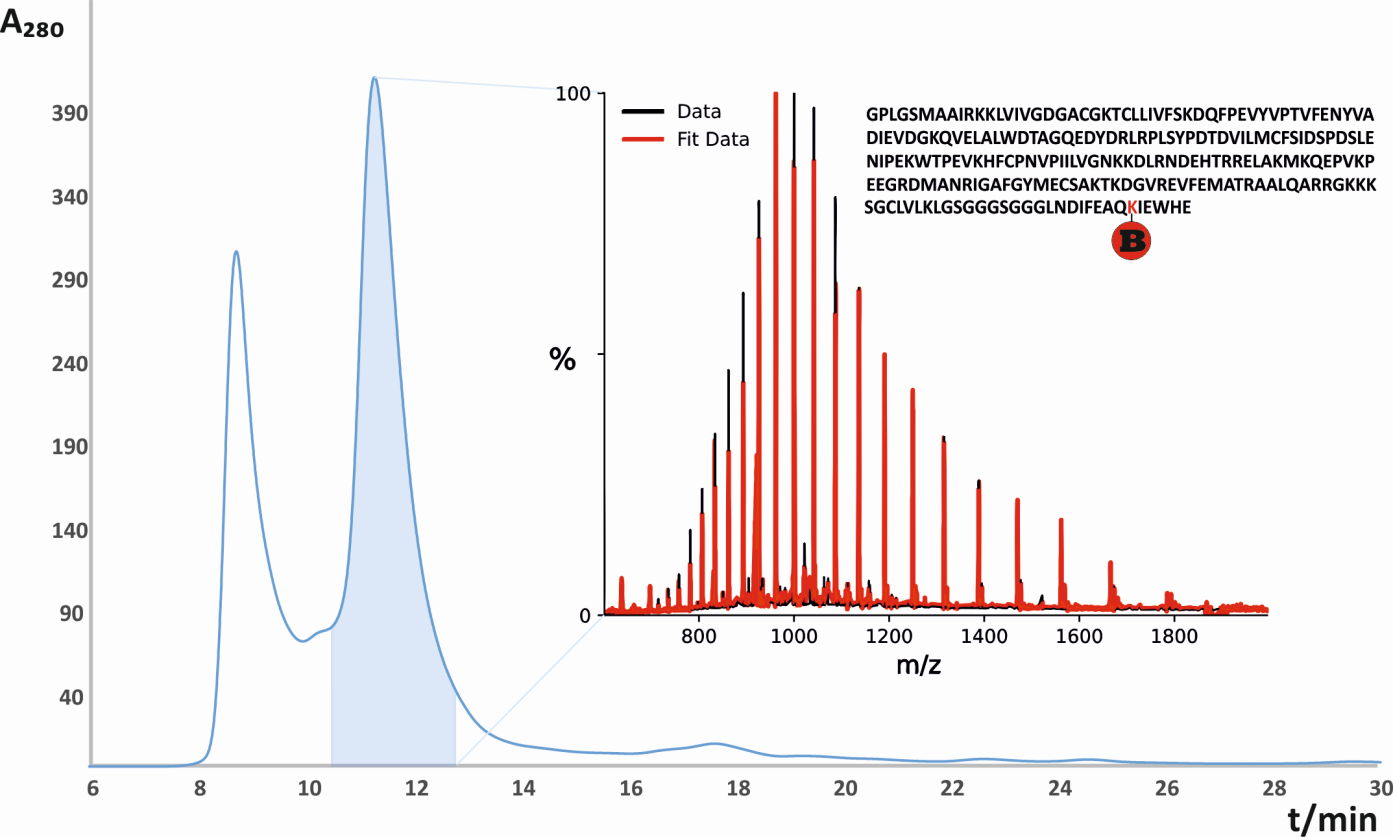
The results of RhoA gel filtration purification. The FPLC chromatogram shows a fraction at the retention time of 11–13 minutes, corresponding to the RhoA protein, as confirmed by Electrospray ionization (ESI) FT-ICR mass spectrometry (inset mass spectra). Fractions that elute between Retention time (RT) 8 and 10 minutes consist of impurities.

After GST-tag removal, the RhoA was purified by the FPLC with a gel filtration column. The peripheral membrane protein nature of the RhoA GTPase suggests that the first fraction (8–10 minutes) in the chromatogram (Fig. 1) could be a RhoA aggregate. The purity of the collected RhoA fraction (11–13 minutes) was evaluated with direct electrospray ionization Fourier-transform ion cyclotron resonance (FT-ICR) mass spectrometry, and the deconvoluted spectra confirmed the presence of the desired RhoA-biotin of desired purity, which could be used for further experiments (Fig. 1).

The measured monoisotopic mass was 24,959.625 Da, corresponding to the theoretical monoisotopic mass with an error of 0.6 ppm. The purified biotin-tagged RhoA protein was further used as a substrate in enzymatic reactions with Toxin B. The biotin tag on expressed RhoA allows the substrate to be bound on the in-house-prepared affinity chips with immobilized NeutrAvidin molecules (20).

### Optimization of the *in situ* enzymatic assay

Several MALDI matrices were tested to achieve the highest ion intensities while minimizing the formation of matrix adducts. The α-Cyano-4-hydroxycinnamic acid (HCCA) matrix, prepared by the published protocol as described in the methods section, was demonstrated to perform better than Sinapinic acid (SA), 2,5-Dihydroxyacetophenon (DHAP), HCCA, ferulic acid (FA), and 2,5-Dihydroxybenzoic acid (DHB) matrices prepared by the standard protocols provided by the manufacturer. Some of the tested matrices formed adducts that interfered with the modified protein signal on the same m/z, and some suffered from low ionization yield of the substrate RhoA protein (Fig. S3). Interestingly, when mixed with stool extract, the immobilized substrate RhoA on the NeutrAvidin chip was more stable than the free RhoA directly added. Additional stability was achieved by adding n-dodecyl-β-D-maltoside (DDM) and Mg^2+^ into the washing buffer and utilizing the protease inhibitor mix (Fig. S4).

**Fig 2:**
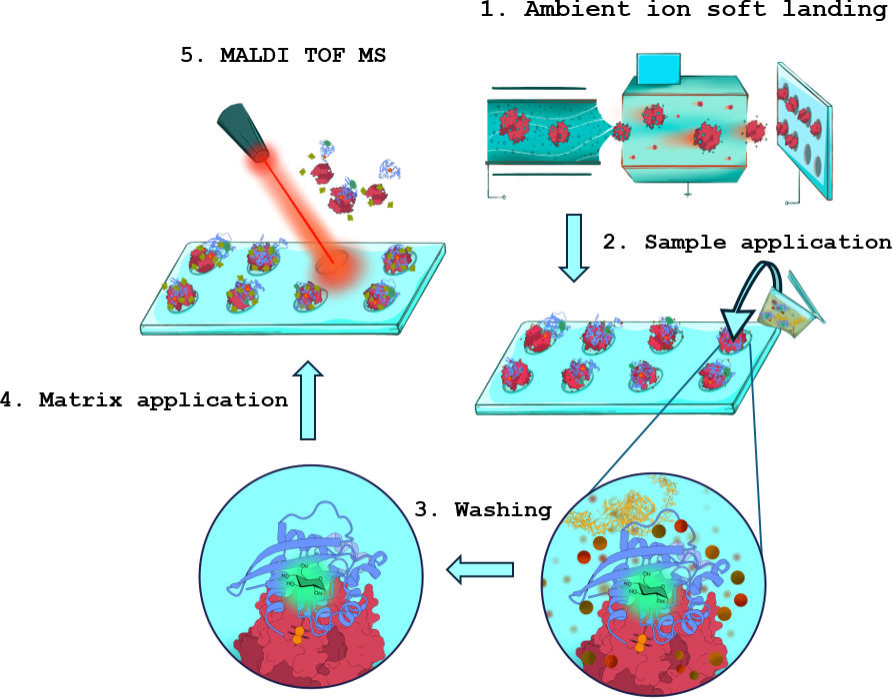
The optimized workflow consists of multiple steps. In the first step, the soft landing is used to prepare the NeutrAvidin chips, followed by the immobilization of the RhoA-biotin substrate on the chip. In the second step, a stool sample in the reaction buffer is applied on the spots with substrate and incubated for 30–80 minutes at 37°C for the substrate to undergo the enzymatic reaction due to the present toxin. The spots with modified/non-modified substrate are washed and overlaid with the HCCA matrix to be measured using the MALDI-TOF-MS.

The experiments showed that reaction time between 30 and 80 minutes allowed the detection of the toxin activity in most stool samples (Fig. S5). The optimized workflow is shown in the following Fig. 2.

### Mass spectrometry detection of Toxin B activity using indirect assay

**Fig 3:**
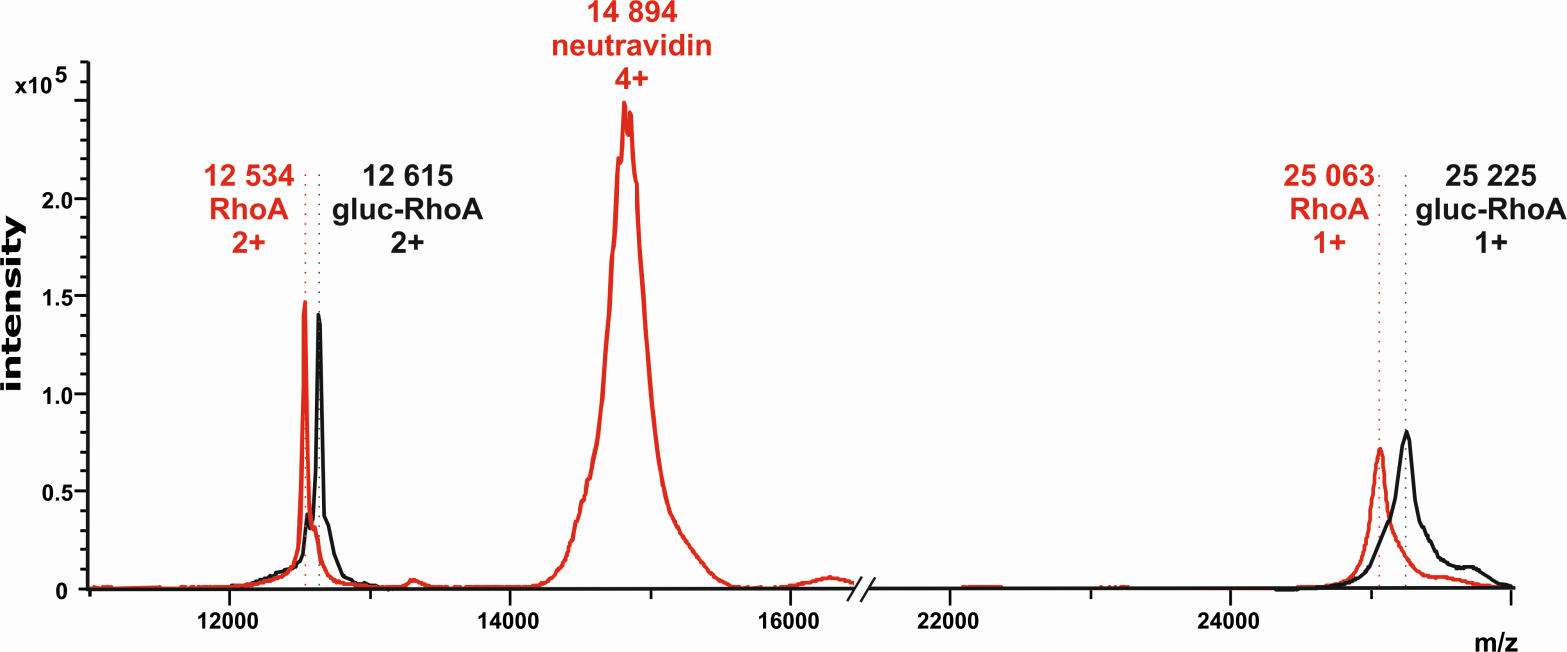
MALDI-MS spectra of intact RhoA (red) and glucosylated RhoA (black) on a NeutrAvidin MALDI chip. Signals obtained for intact RhoA and glucosylated RhoA differ by a mass of 162 (singly charged ions) and 81 (doubly charged ions). The glycosylation of intact RhoA that produces the difference in m/z is a product of Toxin B glucosyltransferase activity. The signal at m/z 14,894 corresponds to quadruply charged NeutrAvidin also released from the surface of the MALDI chip.

The Toxin B presence in the sample based on its activity was monitored by detecting the ion at m/z 25,063 corresponding to intact RhoA protein and the ion at m/z 25,225 corresponding to glucosylated form of RhoA using MALDI-TOF mass spectrometer operated in linear positive mode (Fig. 3).

**Fig 4:**
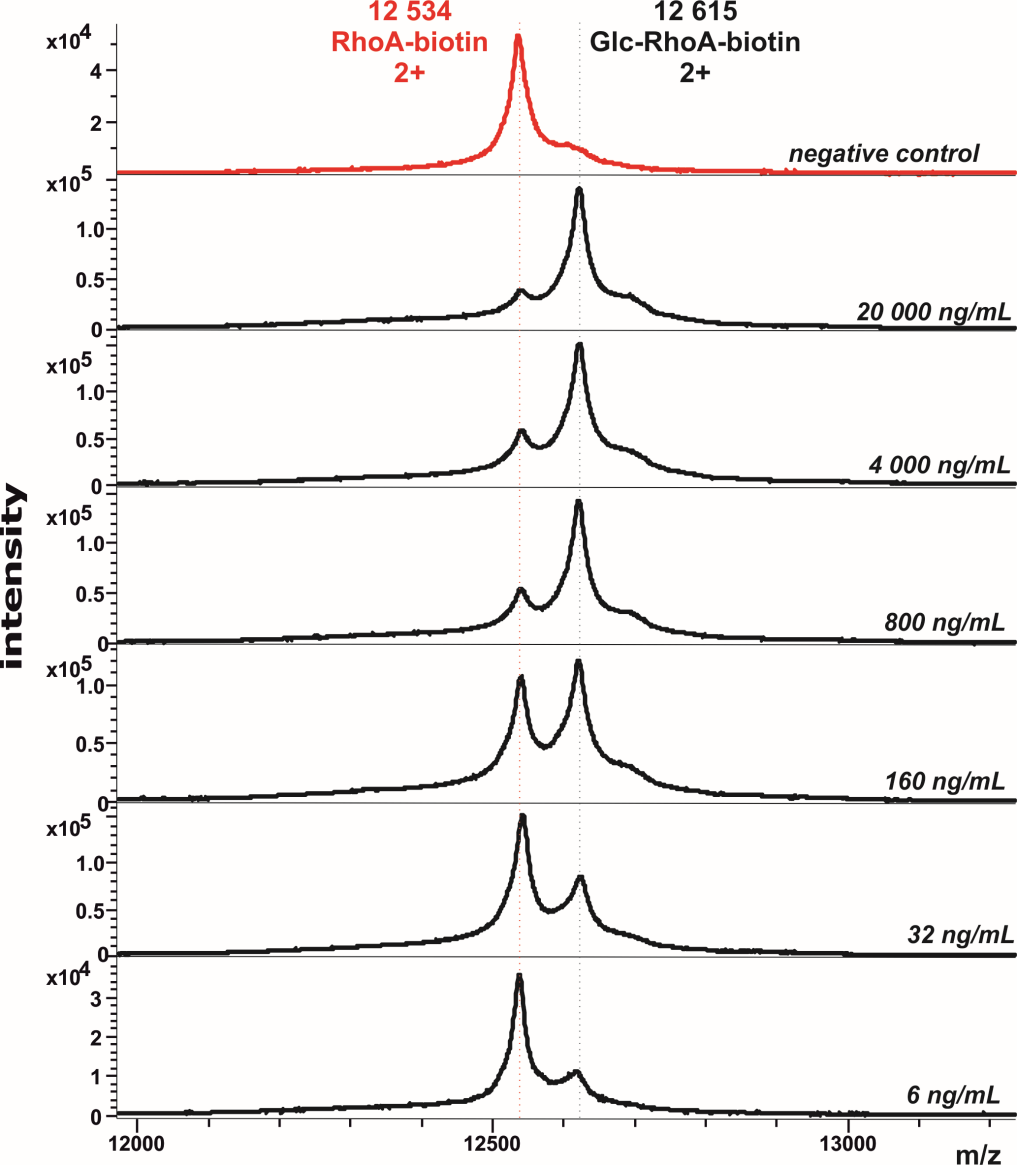
Mass spectra of doubly charged RhoA protein, exposed to stool spiked with different concentrations of recombinant Toxin B in the reaction buffer. The peak of the glucosylated form of RhoA was clearly observed, starting with the Toxin B concentration of 32 ng/mL. The dilution series was measured as a technical pentaplicate on one chip.

To determine the detection limit of our method indirectly assaying the Toxin B activity in the complex matrix (stool), the dilution series of spiked recombinant Toxin B was prepared and used as described in the methods section. The data for each Toxin B concentration represented by signals of glucosylated/non-glucosylated RhoA substrate were obtained using MALDI-TOF mass spectrometry (Fig. 4). The signal intensity of the glucosylated substrate decreases as Toxin B is progressively diluted. For the Toxin B concentration of 32 ng/mL, the signal of the ion corresponding to the glucosylated form of RhoA had an average signal-to-noise ratio of 17. We monitored the signals of the doubly charged ions of both forms of RhoA at m/z 12,534 and 12,615, as they exhibited better resolution and higher intensity compared to the singly charged ions (Fig. 4).

The MALDI spectrum of doubly charged RhoA protein, modified by glucose after treatment with the *C. difficile* strain, is shown in Fig. S6.

The extent of conversion to glycosylated form as defined by the formula in the method section (Formula 1) and corresponding SD and coefficient of variation (CV) was 0.6 (SD = 0.08; CV = 13%) for 32 ng/mL and 0.3 (SD = 0.3; CV = 3%) for 6 ng/mL of spiked Toxin B.

**Fig 5:**
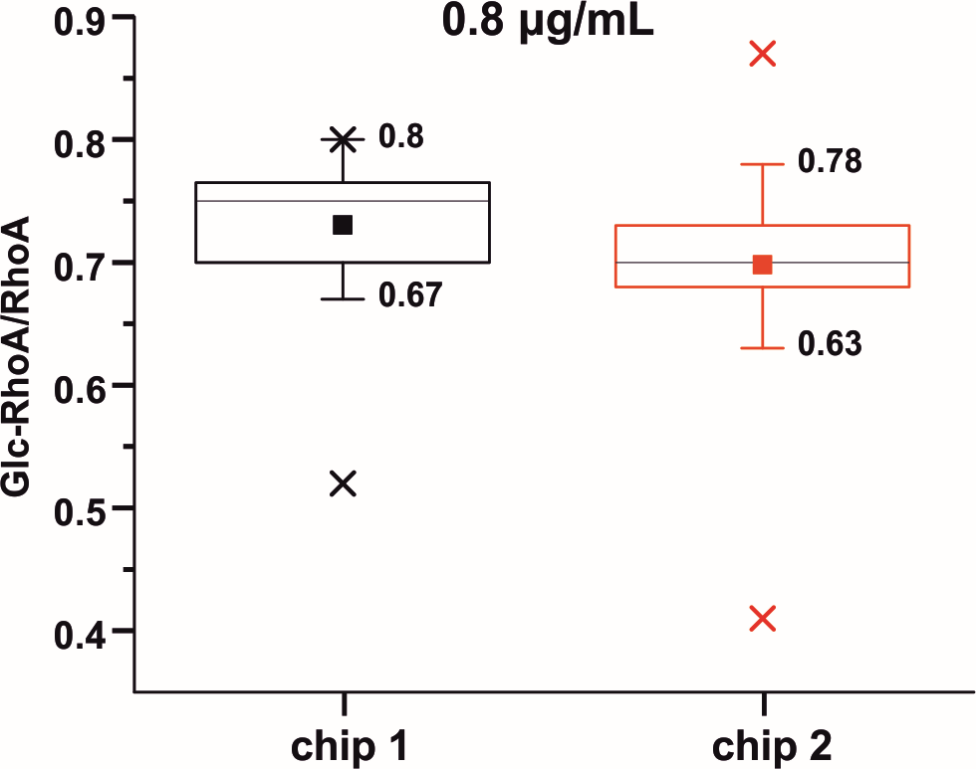
A box plot visualization of the variation test for two NeutrAvidin-modified chips tested using a Toxin B concentration of 0.8 µg/mL. The black box represents the range of values for chip 1 and the red for chip 2. The full square inside the range box represents an average level of modification across the replicate, and the gray line inside the range box represents the median value. The numbers represent minimum and maximum statistically valid extremes, and the cross signs outside the box represent the outlier values. The experiment was performed in 20 technical replicates on each of two different chips, resulting in a total of 40 repetitions.

For a variation test, recombinant Toxin B was spiked into the stool extract at final concentrations of 4 µg/mL and 0.8 µg/mL. The RhoA glucosylation level was calculated as a ratio of glucosylated and RhoA protein signal intensities using the formula described in the method section (Formula 1). Twenty technical replicates were prepared on each of the two chips, resulting in a total of 40 replicates for the statistical evaluation of the method (Fig. 5).

The CV obtained using chip 1 at a Toxin B concentration of 4 µg/mL was 6% (SD 0.05), and at a Toxin B concentration of 0.8 µg/mL, it was 8% (SD 0.06). The CV obtained using chip 2 at a Toxin B concentration of 4 µg/mL was 8% (SD 0.06), and at a Toxin B concentration of 0.8 µg/mL, it was 12% (SD 0.08). The CV between both chips was 7% (SD 0.05) for a Toxin B concentration of 4 µg/mL and 10% (SD 0.07) for a Toxin B concentration of 0.8 µg/mL.

### The use of the optimized assay on CDI-suspected patient samples

The optimized assay was used to detect Toxin B activity in 20 stool samples (Table 1) of patients suspected of CDI (Fig. S5). All samples were tested in the Pilsen Faculty Hospital by IA test that simultaneously detects GDH, Toxin A, and Toxin B or by the loop-mediated isothermal amplification (LAMP) assays for both toxins. The subsequent LAMP assay was performed only if the GDH test was positive in IA but negative for Toxin A and B. Only two patient samples showed these results; thus, there were only two LAMP tests performed (samples #1 and #8). The samples were then shipped to be tested by the presented toxin activity assay based on the MALDI-TOF-MS detection. The MALDI assay was performed in technical duplicates. Details about the assay’s protocols are included in the methods section.

**TABLE 1:** The table represents the results of patient sample tests obtained by standard hospital techniques (GDH and IA against the toxins) and by MALDI assay^*a*^^,^^*b*^^,^^*c*^

Sample number	IA			LAMP			IA/LAMP assay	MALDI assay
	GDH	ToxA	ToxB	GDH	ToxA	ToxB		
1	+	−	−	+	+	+	+	−
2	−	N/A	N/A	N/A	N/A	N/A	−	+
3	+	+	+	N/A	N/A	N/A	+	+
4	−	N/A	N/A	N/A	N/A	N/A	−	−
5	+	+	+	N/A	N/A	N/A	+	−
6	+	+	+	N/A	N/A	N/A	+	+
7	−	N/A	N/A	N/A	N/A	N/A	−	−
8	+	−	−	+	−	−	−	−
9	+	+	+	N/A	N/A	N/A	+	−
10	−	N/A	N/A	N/A	N/A	N/A	−	−
11	−	N/A	N/A	N/A	N/A	N/A	−	−
12	−	N/A	N/A	N/A	N/A	N/A	−	−
13	−	N/A	N/A	N/A	N/A	N/A	−	−
14	−	N/A	N/A	N/A	N/A	N/A	−	−
15	−	N/A	N/A	N/A	N/A	N/A	−	−
16	−	N/A	N/A	N/A	N/A	N/A	−	−
17	−	N/A	N/A	N/A	N/A	N/A	−	−
18	−	N/A	N/A	N/A	N/A	N/A	−	+
19	−	N/A	N/A	N/A	N/A	N/A	−	−
20	−	N/A	N/A	N/A	N/A	N/A	−	−

^*a*^ The check mark indicates positive results. If the GDH test was negative, follow-up testing by IA was not performed. The MALDI assay was performed for every sample, irrespective of the prior GDH results. Only some samples tested positive (patients 1 and 8) by IA were also tested by LAMP.

^*b*^ (+) - positive; (-) - negative; N/A - not applicable.

^*c*^ LAMP - Loop-mediated isothermal amplification; IA - immuno-assay; GDH - glutamate dehydrogenase; ToxA - toxin A; ToxB - toxin B.

**Fig 6:**
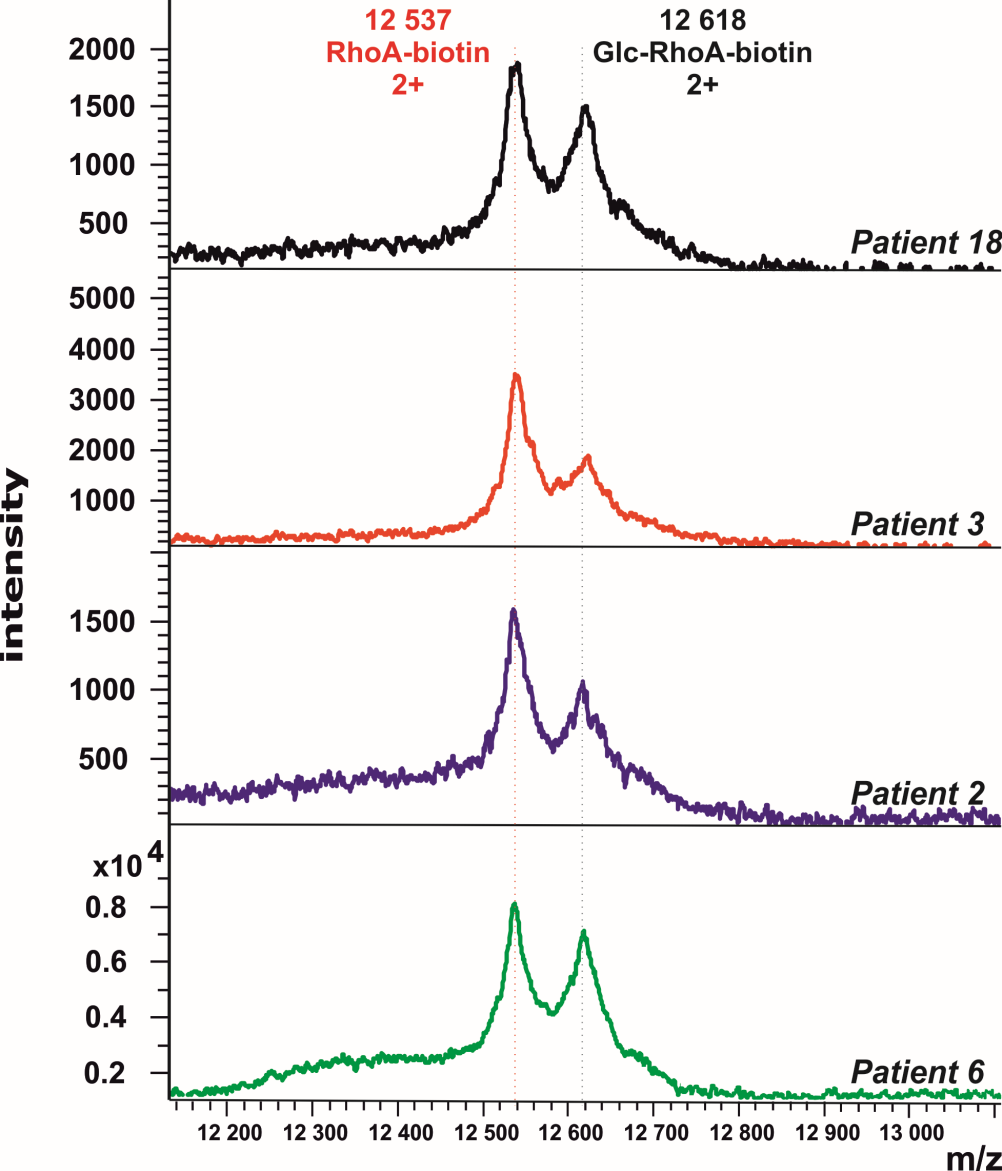
Mass spectra of RhoA substrate for the samples where MALDI assay detected Toxin B (patient samples #2, #3, #6, and #18). The amount of modification in all the samples suggests a possible presence of the active toxin in the sample. The samples were measured in technical duplicates on one chip.

Toxin B activity was detected by MALDI assay in four samples (#2, #3, #6, and #18); two of them agree with the hospital data obtained (#3 and #6). The MALDI-TOF-MS spectra for positively detected samples are in Fig. 6. In the other three samples (#1, #5, and #9), no toxin activity was detected by MALDI, even though the hospital methods provided positive detection.

Table 2 shows the summary of the modification ratios between intact and glycosylated RhoA (extent of RhoA conversion) for four patient samples (#2, #3, #6, and #18) performed in duplicates. The ratio is reproducible within the duplicates, but each of the four samples has a different extent of conversion, which indicates different Toxin B activity in the samples.

**TABLE 2:** Signal intensities for intact and modified RhoA as detected by MALDI-MS in four positive patient samples^*a*^^,^^*b*^

Sample	Replicate	Intensity modified	Intensity non-modified	The extent of conversion (*E*_conv_) (%)
18	1	1,815	2,278	44
	2	8,786	10,949	45
3	1	2,338	3,937	37
	2	3,737	7,841	32
2	1	4,309	6,126	41
	2	1,513	2,015	43
6	1	1,383	1,513	48
	2	10,988	12,101	48

^*a*^ The extent of conversion was calculated according to the formula in the method section and expressed as a percentage. The samples were measured in the technical duplicate.

^*b*^ Intensity modified - intensity of modified substrate; Intensity non-modified - intensity non-modified substrate

## DISCUSSION

Colonization of the gastrointestinal tract by *C. difficile* can happen without the manifestation of typical clinical symptoms of infection. Obtaining positive or negative results only by the highly sensitive genetic NAAT techniques can lead to misidentification of the pathogenesis of the gastrointestinal tract infection and lead to inappropriate therapy. Unfortunately, toxins with similar activity can also be produced by other bacteria, e.g., *P. sordellii*, due to horizontal gene transfer. This is an important mechanism of bacterial evolution (23), especially, but not only, in the spread of antibiotic resistance. This mechanism of bacterial genome plasticity has been described for different virulence genes, and in *Clostridium* spp., horizontal transfer of toxins encoding genes has been documented (32). That is why the determination of free toxins A and B in feces is often performed, especially in CDI-suspected children patients (33), but the results from IA assays do not describe the actual activity of the present toxins. Functional assays oriented on detecting the toxins’ activity, irrespective of the source of microbial infection determined by genomic techniques, could thus significantly improve microbial diagnostics and subsequent therapeutic decisions.

In the present proof-of-concept assay, we demonstrated the ability of MALDI-TOF MS to detect glucosylation of substrate RhoA protein by *C. difficile* Toxin B. The major difficulty of the assay is that it is influenced by the complex matrix of the feces samples. The reaction buffer composition was optimized based on observations made in a previous literature report, where 4-(2-hydroxyethyl)−1-piperazineethanesulfonic acid (HEPES) and Mn^2+^, Mg^2+^, and K^+^ ions were proposed for enhancing the toxin activity (34). The stool samples contain many other enzymatically active proteins; thus, adding an optimized mix of protease inhibitors was a necessity. Metalloprotease inhibitors could not be used due to the need to keep the defined ion strength and composition of the buffer. Strictly from the enzymatic kinetics point of view, the ideal reaction time would be the longest practically possible because, in theory, the longest reaction time leads to the maximum RhoA conversion to the modified Glc-RhoA form. However, the presence of the proteases in the sample matrix limits the lifespan of RhoA in the sample, even if the inhibitors are added to the sample (Fig. S4). Thus, the optimal reaction time is a compromise of these two different factors.

The assay was developed and optimized so that it can be performed *in situ* on the MALDI-compatible chip by applying a stool isolate, which contains the enzymatically active Toxin B. The assay is indirect because it aims to determine the level of glycosylation of RhoA—Toxin B’s substrate—to Glc-RhoA. The RhoA substrate is anchored on the MALDI protein chip by integration between NeutrAvidin immobilized on the chip surface and the biotin tag on the RhoA substrate. This approach overcomes the need for time-consuming cultivation necessary for the routinely used MALDI biotyping and provides information about the actual activity of Toxin B. The optimized assay was tested on the cohort of patient samples and showed results that were for the most part in agreement with the results obtained by the reference methods used in the hospital that provided the samples. Two samples in the cohort (#5 and #9 in Table 1) provided negative results from the new activity assay, although the samples were positive from the IA assay performed in the hospital. Sample #1 was also a false negative, although the hospital detected the toxin by LAMP and not by IA assay. We hypothesize that the different results were caused by the impacted activity of Toxin B in the samples. Thus, assays oriented on measuring the activity and assays oriented on measuring the concentration could not provide the same result. This then manifested as a false negative result, even though the information might mean that the Toxin B in the samples was inactive. Another two samples (#2 and #18) provided positive results by activity assay but were negative in the hospital. It is possible that a toxin with the same activity was present in these samples but originated from other bacterial species and was not detected by IA. The results thus showed two potentially false positive and three false negative results (out of 20 total samples). We speculate that in the case of false negative results, the new activity MALDI-TOF-MS assay was correct, and the Toxin B in the samples was not active at the time of the analysis. It is important to note here that the MALDI-TOF-MS assay is not proposed as a direct replacement for the current methods. It is merely intended as an expansion of the armory of detection tools, which would provide complementary information about the Toxin B activity and thus help to complete the clinical image of the infection severity and of the patient’s condition.

In conclusion, the results show that the presence of virulent *C. difficile* strains can be established based on monitoring products of the Toxin B enzymatic activity in the stool extracts by detecting the level of RhoA protein glycosylation using MALDI-TOF-MS. The assay can be used to detect Toxin B activity in the stool samples of real patients with suspected and confirmed CDI. However, this report is only a proof-of-concept, and further optimization and large-scale evaluation are planned for the follow-up study.

The assay is complementary to other approaches because it provides information about the active Toxin B but not about its total chemical concentration. Another interesting feature of the assays is that by reporting toxin activity, it can identify other toxin-producing bacterial species that obtained the *C. difficile* gene for toxin production through horizontal plasmid transfer (24, 25, 35–37). The main limitation of the assay is the reliance on the stability of RhoA during the assay incubation. Unfortunately, the stability of the RhoA protein in human stool can be impacted by the sample matrix, which is extremely complex. This adversely impacts the assay’s sensitivity, which depends on the incubation time and requires RhoA to be stable enough for the reaction to produce sufficient yields of the glycosylated RhoA variant. Finally, we argue that the implementation of the assay would be relatively easy and economical because the majority of clinical microbiology labs are already equipped with the MALDI-TOF Biotypers that can be used for this assay without any necessary modifications. It does not require cultivation, which makes it very time efficient. The workflow and data interpretation can be easily automated and adjusted for high throughput, thanks to the existing MALDI-TOF and laboratory automation.

## MATERIALS AND METHODS

### Chemicals and materials

Indium tin oxide (ITO)-coated glass slides were purchased from Bruker Daltonics (Massachusetts, USA). Recombinant Toxin B and phosphoramidon were purchased from R&D Systems, Inc. (Minnesota, USA) and NeutrAvidin protein from Thermo Fisher Scientific (Massachusetts, USA). The 10× concentrated cOmplete mini EDTA-free inhibitor cocktail, pepstatin, and bestatin were purchased from Merck (Darmstadt, Germany). All other chemicals were obtained from Merck (Darmstadt, Germany).

Stool samples of 20 patients were provided by the Department of Microbiology, Faculty of Medicine, and University Hospital in Pilsen. The samples were recovered from patients who suffered from diarrhea suspected to be caused by CDI during August and September 2023. All specimens were diagnosed in a routine diagnostic process by GDH, Toxin A, and Toxin B production using fluorescence IA and interpreted using Standard F2400 reader as recommended by the manufacturer (Lab Mark, Prague, Czech Republic). The inconclusive IA resulting samples (GDH positive, toxins negative) were simultaneously alarmed using LAMP for *gdh*, *tcdA* (ToxA), and *tcdB* (ToxB) genes (eazyplex *C. difficile* Kit, AmplexDiagnostics GmbH, Gars-Bahnhof, Germany). Based on this combination of tests, five samples were identified as positive for *C. difficile*.

### Manufacturing of affinity MALDI-compatible chips

A previously described apparatus for ambient ion soft landing (38) was used to prepare NeutrAvidin affinity chips on ITO glass slides. The geometry of the deposited NeutrAvidin array on the ITO glass was defined using sticker masks. The 0.6 mg/mL NeutrAvidin solution in 20 mM ammonium bicarbonate buffer, pH 7.8, was delivered by the syringe pump at a flow rate of 60 µL/h using 100 µm fused silica capillary connected to the micro-spray emitter with an applied voltage of positive 1.5 kV to create an electrospray. A stream of preheated nitrogen gas (50°C–60°C) supported the nebulization and evaporation of droplets. After the soft landing, a washing step with deionized water was performed to remove any excess material, allowing only NeutrAvidin bound to the surface to remain.

### Cultivation of *C. difficile*

Isolates of *C. difficile* were cultivated on *C. difficile* Selective agar (Oxoid, Hants, UK) in an anaerobic atmosphere developed by the Anaerocult system (Merck, Prague, Czech Republic) at 37°C for 48 hours. For toxin detection, colonies identified using the MALDI Biotyper Sirius System (Bruker Daltonics GmbH, Bremen, Germany) were used for inoculation of Oxoid Fastidious Anaerobe Broth (Oxoid, Hants, UK) and incubated in an anaerobic atmosphere for another 24 hours. Bacteria were collected by centrifugation (12,000 g, 2 minutes) and used for further experiments.

### Biotin-tagged RhoA recombinant protein expression

The substrate RhoA-biotin was prepared as described by (39, 39) with several modifications leading to the production of recombinant RhoA protein with a biotin tag. A human RhoA sequence was modified with the avi-tag linker necessary for biotinylation by BirA biotin ligase. The pGex-6p-1 expression plasmid was used as the final construct (Fig. S1), and the insert (Fig. S2) was used to produce the biotin-tagged GST-RhoA fusion protein. The expression was made in the Luria-Bertani (LB) media using the BL-21 Bir. A producing *E. coli* with a biotinylation ability under Isopropyl β-D-1-thiogalactopyranoside (IPTG) induction control. When the OD_600_ reached 0.9, the IPTG and the biotin were added and left to incubate overnight at 18°C. The protein was purified using GST-Sepharose. The GST-hRHoA-Avi-tag product was cleaved overnight on the column at 6°C using a PreScission protease. Purified protein was aliquoted into a 1 mg/mL stock solution and stored at −80°C.

### *In situ* enrichment of biotin-tagged RhoA

One microliter of RhoA with a biotin Tag at a concentration of 0.5 mg/mL was applied on each spot of the affinity MALDI chip modified with NeutrAvidin and incubated in a humidity chamber for 60 minutes. The incubation was followed by a fast wash with a buffer solution containing 20 mM HEPES, 50 mM NaCl, 10 mM MgCl_2_, 1 mM tris(2-carboxyethyl) phosphine (TCEP), 0.17 mM DDM, pH 7.5, and consecutive wash with deionized water. The chip was dried at room temperature. The total amount of substrate protein deposited was 20 pmol.

### *In situ* enzymatic reaction with recombinant Toxin B

The following protocol prepared samples for the assay optimization and evaluation. The feces specimen was diluted to the final concentration of 1% (wt/wt) in 20 mM HEPES, 100 mM KCl, 10 mM MgCl_2_, 10 mM MnCl_2_, 1 mM TCEP, and 0.17 mM DDM, with protease inhibitors cocktail (cOmplete Mini EDTA free, pepstatin, phosphoramidon, and bestatin) reaction buffer, pH 7.5. Recombinant Toxin B was added into the feces at different concentrations as a 5× dilution series from 20–0.006 µg/mL. The dilution series was measured on one chip in a technical pentaplicate.

One microliter of each sample with a different concentration of Toxin B was applied on the MALDI surface with immobilized RhoA protein serving as a substrate. After 1 hour of incubation at 37°C, the affinity chip was washed three times with a washing buffer and twice with deionized water and let dry at room temperature. Each spot was overlaid by an HCCA prepared as a saturated solution in formic acid, acetonitrile, and water mixed in the 1:2:3 ratio as described previously by Cohen et al. (40).

The optimized assay was tested on the CDI-suspected patient samples obtained from Pilsen Faculty Hospital. The samples were diluted with the same HEPES reaction buffer as previously to provide a 1% solution, but no recombinant Toxin B was added. Twenty patient samples were analogically applied on the NeutrAvidin chip with immobilized substrate RhoA in technical duplicates of 1 µL of stock solution per spot.

### MALDI mass spectrometry and data processing

The samples from MALDI chips were detected using an Autoflex speed MALDI-TOF mass spectrometer (Bruker Daltonics) equipped with a Smart beam-II laser and operated in the positive linear mode. A partial sample random walk and accumulation of 7,000 laser shots for each spot were set up for data acquisition. The MS data were acquired in the *m/z* range of 6,000–300,000 Th. The laser frequency was set to 500 Hz, and the detector gain was set to 2,860 V.

The method was evaluated using two different Toxin B concentrations spiked into the stool isolate using two different affinity chips. Samples at 4 and 0.8 µg/mL concentrations were applied on 20 spots in the technical replicate of 1 µL per spot on two different chips, resulting in 40 repetitions of both concentrations. The extent of conversion (*E*_conv_) of the RhoA substrate to its glycosylated form was calculated as the following ratio:


Formula 1 $$E_{conv}=\frac{I_{glyco}}{I_{intact}+I_{glyco}}$$

Where *I*_glyco_ is the signal intensity of modified RhoA protein in the MALDI MS spectrum, and *I*_intact_ is the signal intensity of intact RhoA in the same spectrum.

## Data Availability

The raw mass spectrometry data were deposited under ProteomeXchange Consortium via the PRIDE partner repository with the data set identifier PXD055622 (41).

## SUPPLEMENTAL MATERIAL

Fig. S1 spectrum.02453-24-s0001.tif Scheme of plasmid and insert.

Fig. S2 spectrum.02453-24-s0002.tif Plasmid insert sequence.

Fig. S3 spectrum.02453-24-s0003.tif Comparison of the resulting spectra during the MALDI matrix optimization experiments.

Fig. S4 spectrum.02453-24-s0004.tif RhoA MALDI mass spectra without and with protease inhibitors.

Fig. S5 spectrum.02453-24-s0005.tif MALDI mass spectra of RhoA protein from all 20 patient samples.

Fig. S6 spectrum.02453-24-s0006.tif MALDI spectrum of doubly charged RhoA protein modified by glucose.

Legends spectrum.02453-24-s0007.docx Legends for supplemental figures.

ASM does not own the copyrights to Supplemental Material that may be linked to, or accessed through, an article. The authors have granted ASM a non-exclusive, world-wide license to publish the Supplemental Material files. Please contact the corresponding author directly for reuse.
