# Supplementary figures and images for: Proof-of-concept MALDI-TOF-MS assay for the detection of Toxin B enzymatic activity in Clostridioides difficile infection
Source: Microbiol Spectr. 2025 Mar 31;13(5):e02453-24. doi: 10.1128/spectrum.02453-24 (PMC12054005; doi:10.1128/spectrum.02453-24)

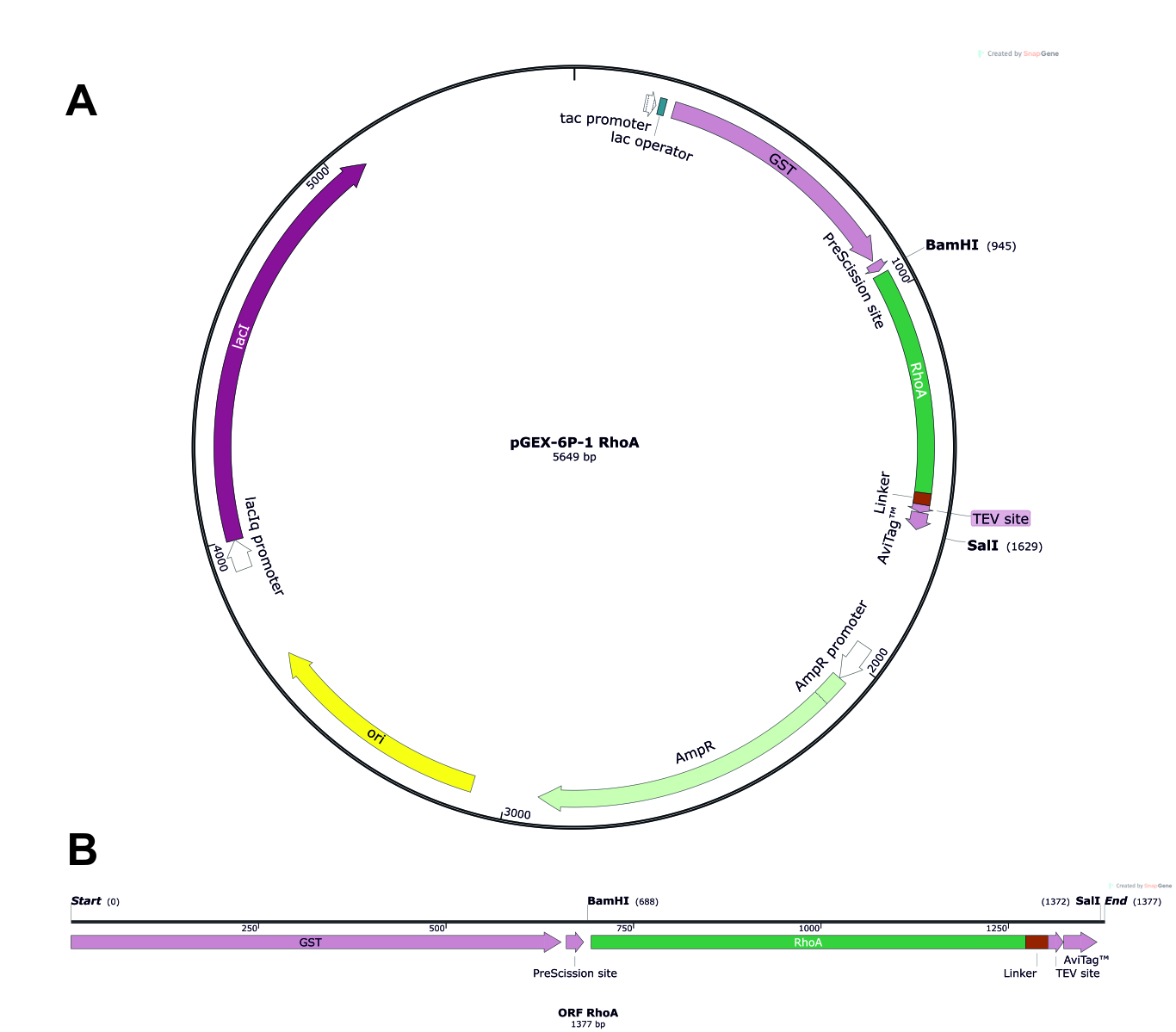

Supplement: Fig. S1 — Scheme of plasmid and insert. [file spectrum.02453-24-s0001.tif]

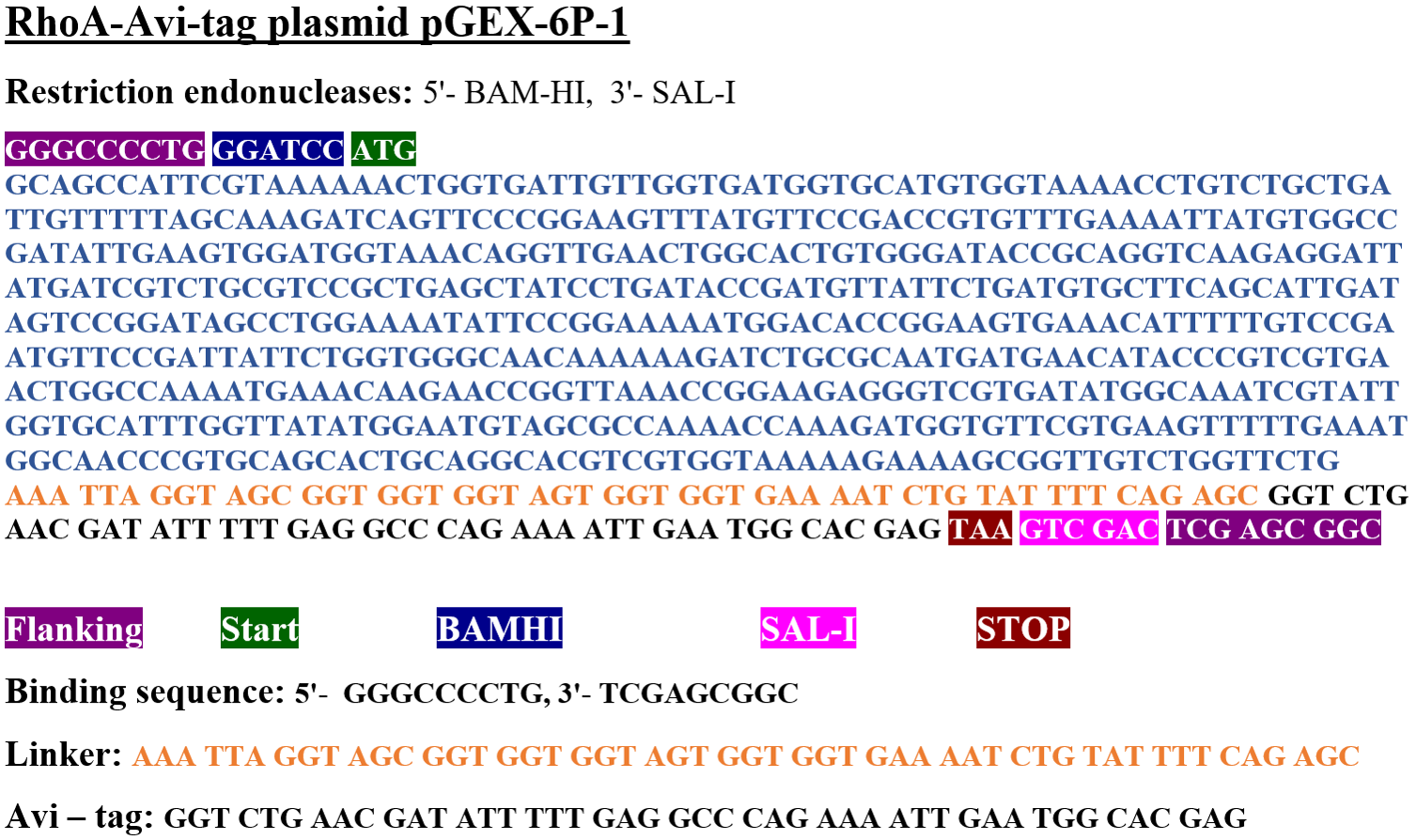

Supplement: Fig. S2 — Plasmid insert sequence. [file spectrum.02453-24-s0002.tif]

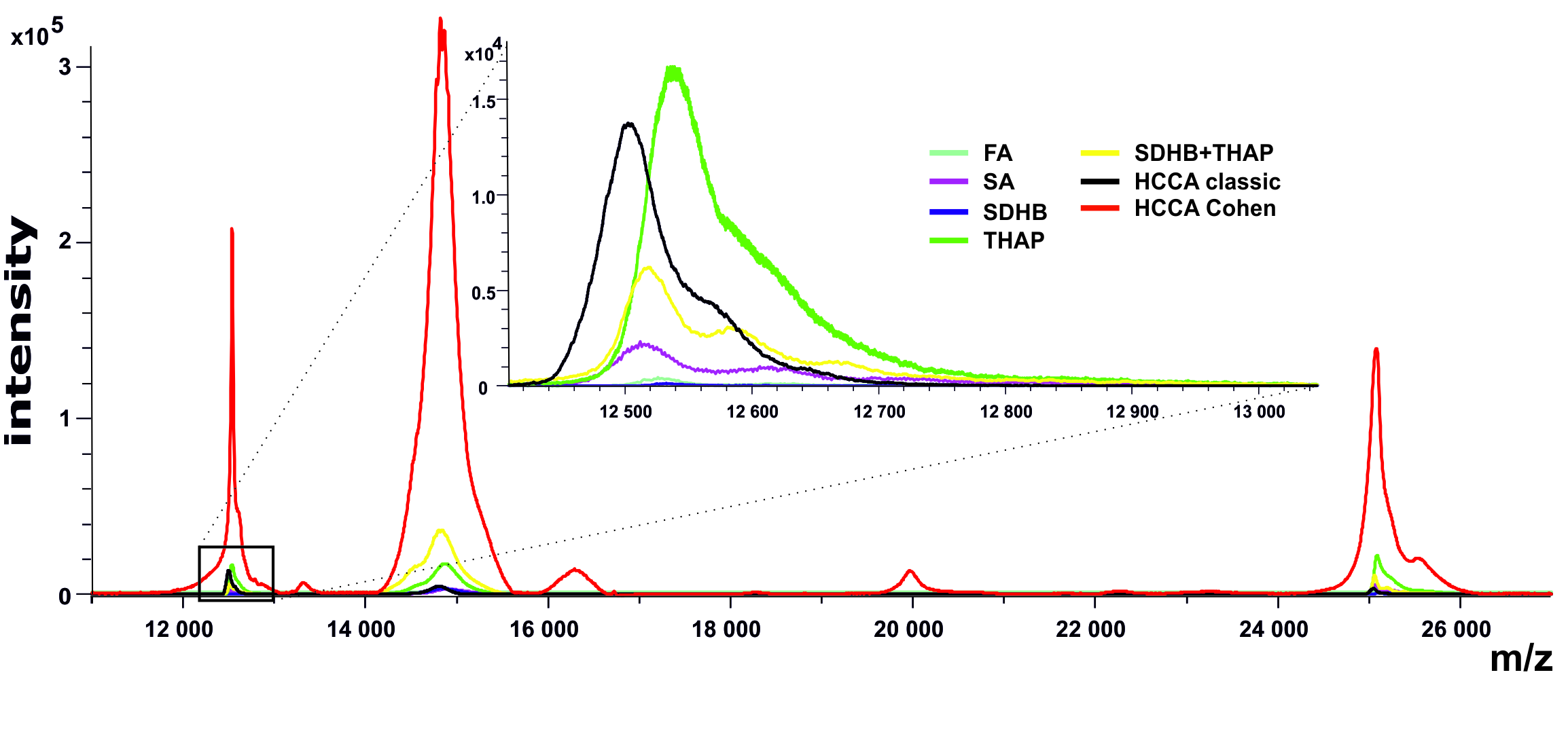

Supplement: Fig. S3 — Comparison of the resulting spectra during the MALDI matrix optimization experiments. [file spectrum.02453-24-s0003.tif]

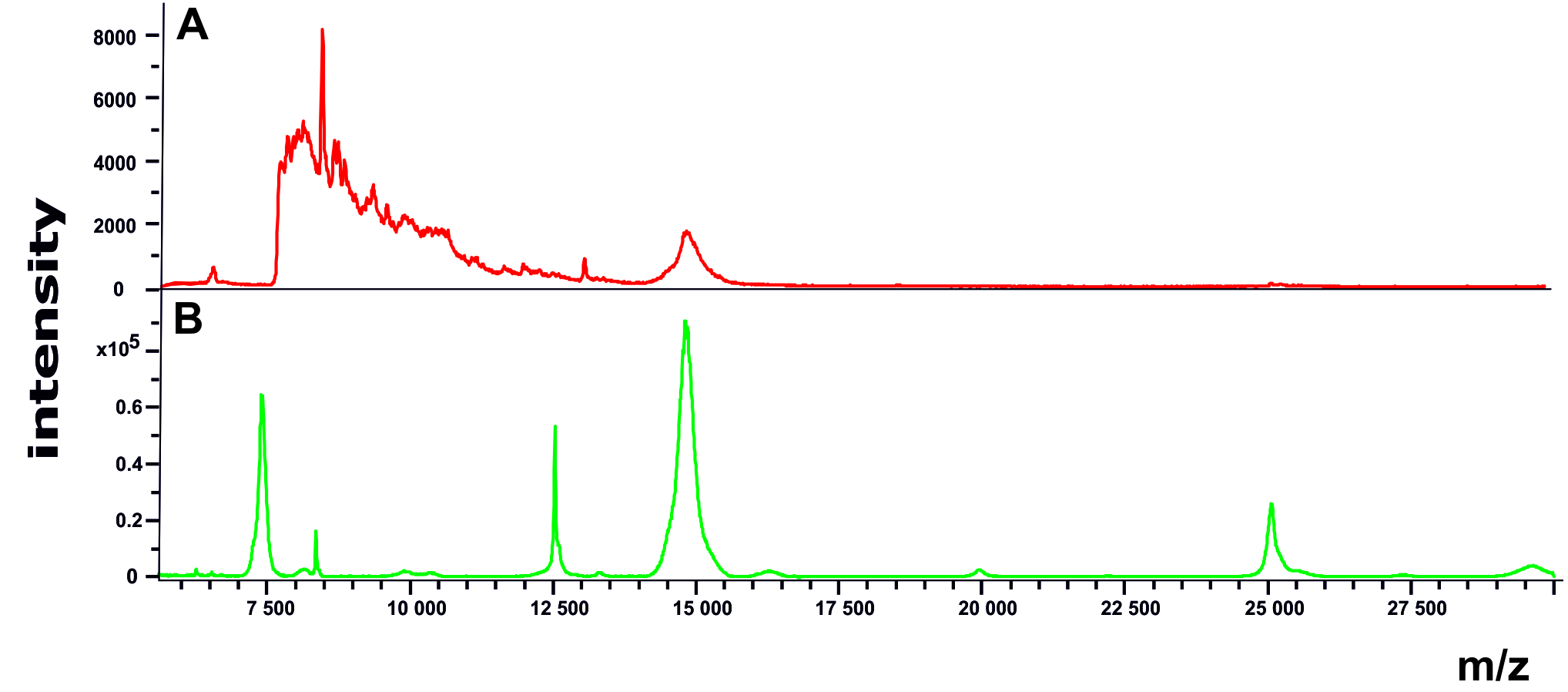

Supplement: Fig. S4 — RhoA MALDI mass spectra without and with protease inhibitors. [file spectrum.02453-24-s0004.tif]

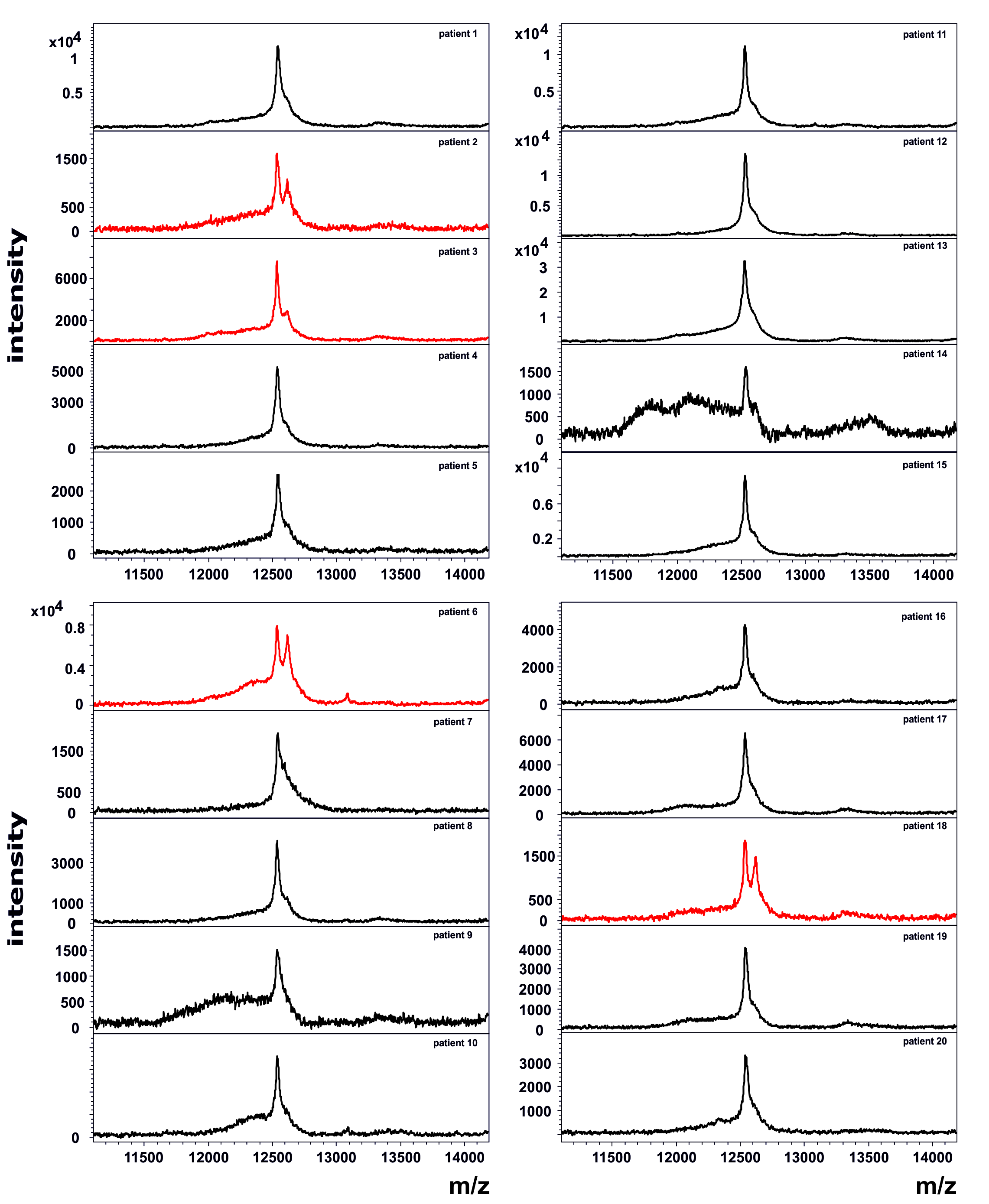

Supplement: Fig. S5 — MALDI mass spectra of RhoA protein from all 20 patient samples. [file spectrum.02453-24-s0005.tif]

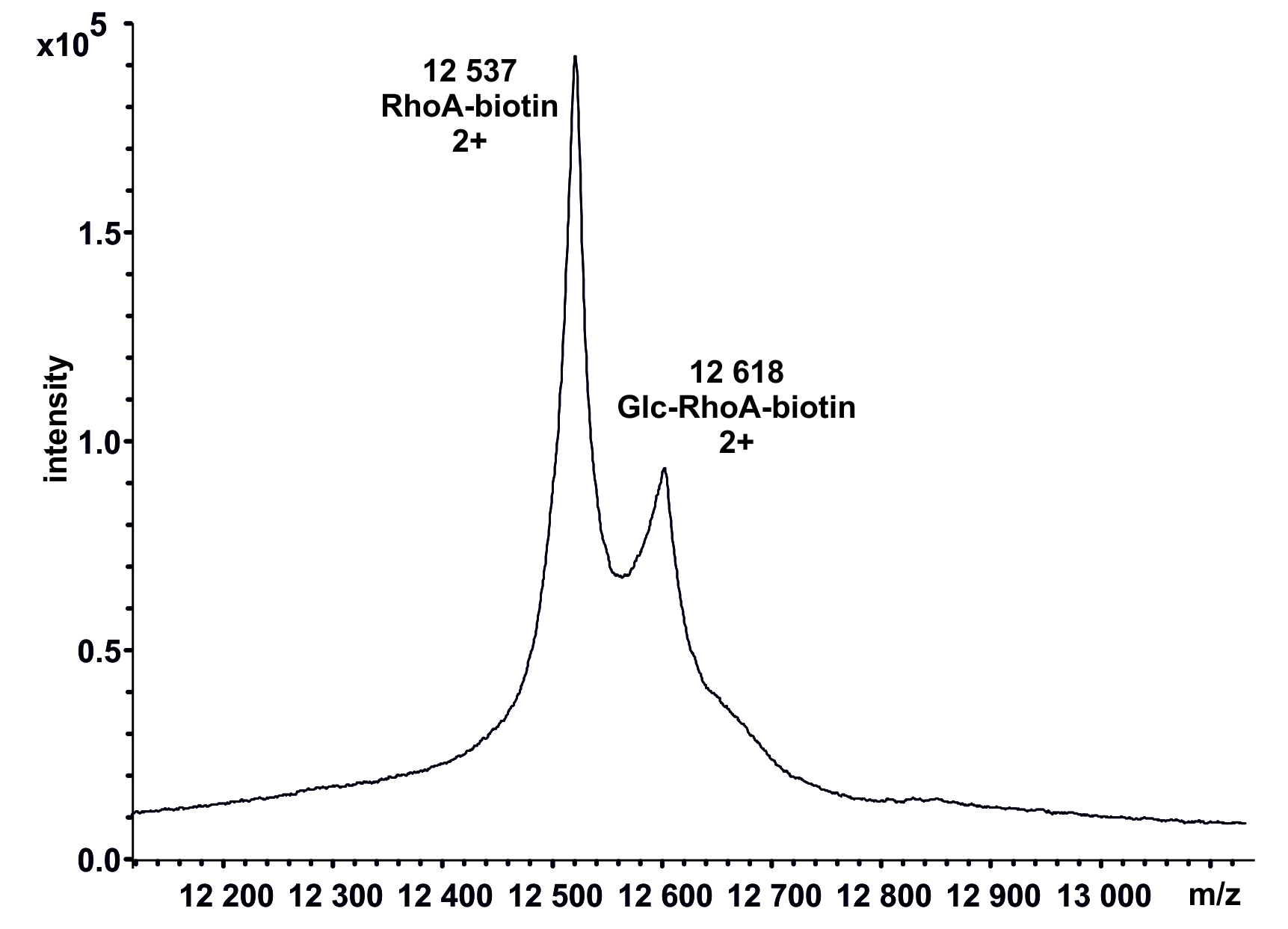

Supplement: Fig. S6 — MALDI spectrum of doubly charged RhoA protein modified by glucose. [file spectrum.02453-24-s0006.tif]
