# Supplementary material for: Proof-of-concept MALDI-TOF-MS assay for the detection of Toxin B enzymatic activity in Clostridioides difficile infection
Source: Microbiol Spectr. 2025 Mar 31;13(5):e02453-24. doi: 10.1128/spectrum.02453-24 (PMC12054005; doi:10.1128/spectrum.02453-24)
Supplement: Legends — for supplemental figures. [file spectrum.02453-24-s0007.docx]

**SUPPLEMENT****AL DATA**

Figure S 1 - **A**) Scheme of the plasmid and **B)** Scheme of the insert. The detailed sequence is shown in Figure S 2.

Figure S 2 – The plasmid insert sequence used for the recombinant expression of the substrate RhoA.

Figure S 3 – A comparison of the resulting spectra during the MALDI matrix optimization experiments. It is possible to detect the singly and doubly charged RhoA ions, and the picture zooms on the doubly charged RhoA ion, providing overall better intensity and resolution than the singly charged ion. The SA provides an adduct that interferes with the targeted protein modification. Therefore, the most suitable matrix is HCCA, as described by Cohen et al. as described in the methods section.

Figure S 4 – RhoA MALDI mass spectra without the addition of protease inhibitors (A) and after the addition of protease inhibitors (B). Measured with the optimized condition and the optimized HCCA matrix.

Figure S 5 –MALDI mass spectra of RhoA protein from all 20 patient samples. Samples where the modified form of RhoA was detected are displayed in red. The mass difference in the positive samples represents the substrate glucosylation due to the influence of the ToxinB in the sample. The samples were measured as technical duplicate.

Figure S 6: MALDI spectrum of doubly charged RhoA protein modified by glucose after the treatment with strain provided for external quality control by Czech National Institute of Public Health. The cell culture was performed as discussed in the methods section.
